# 45 Years of Research on Psychotherapy for Depression: Lessons for the Future

**DOI:** 10.1192/j.eurpsy.2022.40

**Published:** 2022-09-01

**Authors:** P. Cuijpers

**Affiliations:** Vrije Universiteit Amsterdam, Department of Clinical, Neuro and Developmental Psychology, Amsterdam, Netherlands

## Abstract

More than 800 randomised controlled trials have examined the effects of psychotherapies for depression and compared psychotherapies with control conditions, with each other, with pharmacotherapy and with combined treatments. These trials have also examined the effects of therapies in specific target groups, such as women with perinatal depression, children and adolescents, older adults, people with general medical disorders and many others. Furthermore, the effects have not just been examined on depressive symptoms, but also on other outcomes, such as quality of life, functional limitations and social support. In this presentation I will present the results of a large meta-analytic project in which new trials are continuously added. I will show that the most important therapies are effective, that most therapies have comparable effects, that these effects remain significant up to one year follow up and that the therapies are effective in most specific groups. But meta-analyses should also be considered with caution, because they overestimate the effects of therapies. The effects of therapies are comparable to those of pharmacotherapy, but at the longer term psychotherapies are more effective. Combined therapy is more effective than either one alone, at the short and longer term.

**Disclosure:**

No significant relationships.

